# Greater and More Focused Measures Are Needed to Tackle Diabetes and Obesity Epidemics in the Nations of the Gulf Cooperation Council

**DOI:** 10.1155/2021/6661346

**Published:** 2021-03-26

**Authors:** Abdelilah Arredouani

**Affiliations:** ^1^Diabetes Research Center; Qatar Biomedical Research Institute, Hamad Bin Khalifa University, Doha, Qatar; ^2^College of Health and Life Sciences, Hamad Bin Khalifa University, Doha, Qatar

## Abstract

Beyond the suffering of the affected subjects, type 2 diabetes (T2D) and obesity epidemics gripping the Gulf Cooperation Council (GCC) states are expected to seriously jeopardize these nations' economies and development due to productivity losses. Available data show that healthcare budgets in GCC nations are under tremendous pressure because of diabetes- and obesity-linked comorbidities. Furthermore, T2D, once an over-forties disease, risks becoming a whole-adult-life condition because of obesity-associated early-onset T2D and prediabetes. The incidence of T2D is set to worsen unless efficient actions are taken to fight obesity and prevent the conversion of prediabetes to T2D. There is a consensus that the concomitant increase in obesity rates drives T2D rates upward. Fighting obesity at all levels should, therefore, take center stage for the GCC nations. The battle against obesity and T2D is a long-term and complex one. Therefore, only through concerted efforts between several public and private actors, including health, economic, and urbanization agencies, food producers and retailers, schools, families, youth organizations, sports clubs, and voluntary organizations, can this battle be won. The present review tries to assess the current status of diabetes and obesity epidemics in the GCC context and take stock of some of the policies and initiatives that have been, or need to be, implemented to address their growing burden.

## 1. Introduction

Diabetes is a global public health burden. In 2019, 463 million individuals (9.3%) had diabetes globally, and this number is expected to reach 700 million (10.9%) by 2045 [1]. Of significant concern is the fact that 1 in 2 people (232 million) with diabetes is undiagnosed [1], reducing the odds of preventing or at least delaying the diabetes' comorbidities. Further, diabetes increases the risk of many other illnesses, including many cancer types [2], Alzheimer's, and Parkinson's diseases [3, 4]. Additionally, some 374 million individuals (7.5%) had prediabetes in 2019. This rate is projected to reach 8.6% (548 million) by 2045 [1]. Annually, 5 to 10% of prediabetes patients progress to T2D [5, 6], making prediabetes a significant risk factor for T2D. Consequently, T2D incidence will worsen if prediabetes progression is not prevented.

Although the role of genetic predisposition to diabetes cannot be overlooked [7], it is widely accepted that it is primarily the skyrocketing obesity prevalence that explains the unwavering increase in the incidence of T2D worldwide [8]. In 2016, about 650 million were obese worldwide (BMI ≥ 30 kg/m^2^) [9]. Obesity causes several comorbidities besides T2D, including insulin resistance, hypertension, dyslipidemia, cardiovascular disease, stroke, sleep apnea, nonalcoholic fatty liver disease [10], and numerous cancers [11]. Diabetes' impact on health budgets is undeniable. In 2019, the world spent $760 billion directly on diabetes, and this figure is expected to reach $845 billion by 2045 [12]. Moreover, prediabetes and undiagnosed diabetes place further pressure on health budgets. In 2017, USA spent $31.7 billion and $43.4 billion on undiagnosed diabetes and prediabetes, respectively [13]. On the other hand, with its numerous associated comorbidities, the global economic impact of obesity is roughly $2 trillion annually [14, 15]. The health and socioeconomic impacts of diabetes and obesity call for urgent actions to tackle these relentless epidemics.

## 2. Diabetes in the GCC: The Top Health Priority

The GCC countries, Bahrain, Kuwait, Oman, Qatar, Kingdom of Saudi Arabia (KSA), and the United Arab Emirates (UAE), have populations that are among the least protected against T2D. It has now become axiomatic that there is an alarming diabetes epidemic sweeping these countries. In KSA, for example, the number of people with diabetes increased from 0.9 million in 1992 to 2.5 million in 2010 [16]. A 1982 study involving 1387 males from the Al-Kharj area of central KSA showed that 2.5% had T2D [17], while another study from the same area found that 30% of participants (*n* = 6024) had T2D in 2009 [18]. Similarly, in Kuwait, the prevalence of T2D increased from 7.6% in 1990 [19] to 14.8% in 1996 [20]. Further, remarkable T2D prevalence of 50.6% was reported in a 2010 cross-sectional household survey of 2,487 Kuwaiti nationals (>50 years) [21]. The concerning T2D rates in the GCC are expressed in the 2019 IDF statistics, where five countries, KSA (18.3%), Kuwait (22%), Qatar (15.5%), Bahrain (16.3%), and the UAE (15.4%), ranked among the 20 countries with the highest prevalence of diabetes worldwide (18 to 99 years). The five nations' average prevalence is 17.5 ± 2.8%, which is almost double the global prevalence (9.3%). Oman is relatively less affected at 8% [1, 22]. However, these rates should be taken with caution because much higher rates were reported. For instance, prevalence of 31.6% was reported in KSA's central region [23]. Likewise, higher T2D rates were reported for Qatar (23%) [24] and the UAE (25%) [25]. The situation might be worse in Kuwait, where a recent study (*n* = 3915) revealed that 27.4% of obese (BMI ≥ 30 kg/m^2^) and 36% of hypertensive adults had T2D [26]. Furthermore, numerous GCC studies indicate that about 50% of T2D individuals are undiagnosed [25, 27–29]. It is also worth noting that the recent IDF data indicate that diabetes' prevalence in subjects below 20 and 40 years of age in the MENA region is almost double the prevalence in Europe and triple that in Africa, suggesting an early onset of diabetes in the MENA region and thus the need for early diabetes screening programs. In terms of economic impact, T2D places enormous pressure on GCC health budgets. For instance, Qatar, UAE, and Bahrain have spent, respectively, $0.5bn, $2bn, and $0.27bn directly on T2D diabetes in 2015 [30, 31]. Likewise, in KSA, 34% ($10bn) of the 2014 health budget ($29 billion) was allocated to diabetes treatment. Given the number of undiagnosed diabetes cases, the actual expenditure is likely higher. The average annual cost per person with diabetes, excluding nonquantifiable costs such as pain, suffering, care by family members, and health providers' training, ranges from $752 in Oman to $1751 in Qatar [32]. Moreover, like elsewhere, diabetes affects the GCC states' economies due to diabetes-associated productivity losses consequent to absenteeism, unemployment, disability, and early mortality. Unfortunately, economic studies that estimate the real impact of diabetes on economic productivity in GCC are very scarce. For example, a recent study from Bahrain [30] estimated at $3.62 million the cost due to diabetes-associated absenteeism in 2015, when the number of subjects with diabetes was 154300 (IDF Atlas, 2015). With 202700 subjects with diabetes today (IDF, February 2020), this estimation has likely increased. By extrapolation, in a country like KSA, where 4,275,200 people have diabetes (IDF, February 2020), productivity losses due to diabetes must be several times higher than those in Bahrain.

### 2.1. Prediabetes in the GCC: An Epidemic to Watch Closely

Though scarce, studies from the different GCC states have reported high rates of prediabetes. In Kuwait, a 2014 study on adults (*n* = 3915) found a prediabetes rate of 19.4% [26], while Zhang and colleagues found that 40% of adults (*n* = 960) had prediabetes in a 2016 study [33]. Likewise, the SAUDI-DM study in KSA (*n* = 18034, >30 years) found that prediabetes affected 25.5% of the total sample [34]. Prediabetes affected 27.6% of Saudi adult men in a more recent study (*n* = 381) [35]. In the UAE, the 2017 Dubai Diabetes Survey revealed that 18.6% of adult Emiratis had prediabetes [36]. In Qatar, a pilot screening program (*n* = 3500) involving individuals at risk for T2D (obese, hypertensive, family history of diabetes) revealed that about 25% of the screened individuals had prediabetes (Ministry of Public Health presentation, 2016). In Oman, a 2011 study (*n* = 1300) found prevalence of 35% [37]. Given the annual conversion rate from prediabetes to T2D (5–10%) [5, 6], the high prediabetes' prevalence described above raises real concerns in the GCC nations and calls for immediate actions.

### 2.2. Childhood T2D in GCC: A Threat to Future Generations

T2D in children, also called early-onset T2D, has become a global challenge [38, 39]. For instance, fewer than 3% of all cases of new-onset diabetes in adolescents were attributed to early-onset T2D in USA until 2001, while 45% of cases in 2011 were accounted for by early-onset T2D [40, 41]. Similar trends were reported from many other countries worldwide, including GCC nations. In the UAE, for example, a study on 1034 overweight/obese Emirati students aged 11 to 17 years found that 5.4% and 0.87% had prediabetes and T2D, respectively [42]. In Qatar, the incidence of childhood T2D increased from 1.82/100,000 in 2012 to 2.7/100,000 in 2016 [43]. In KSA, the SAUDI-DM (*n* = 23523; age ≤18 years) revealed that, in 6–18-year-old children (*n* = 17207), the prevalence of prediabetes was 6.12%, while newly identified cases of T2D represented 4.27% [34]; this is one of the highest documented T2D diabetes rates for this age group. Worryingly, 90% of the children and adolescents with diabetes were unaware of their disease [34]. Another major concern with childhood T2D is that, unlike adult T2D, the decline in pancreatic *β*-cell function is 3- to 4-fold faster, and the rate of therapeutic failure in young people is significantly higher than that in adults [44]. The underlying molecular mechanisms are still to be deciphered. Consequently, efforts should be focused on strategies to prevent childhood T2D and prediabetes in the first place. If GCC citizens continue developing chronic disorders at a young age, these countries' growth will undoubtedly be jeopardized.

### 2.3. Diabetes in the GCC: Who Is to Blame?

Type 2 diabetes is multifactorial. While one cannot exclude an increased genetic predisposition of GCC populations to T2D, it is recognized that T2D and prediabetes epidemics in the region are primarily attributed to the concomitant obesity epidemic. The prevalence of obesity in adults in most GCC countries is higher than 30% [45]. In Qatar, the 2019 Qatar Biobank's annual report (*n* = 18,000 adults) showed that 35% were overweight, and 43% were obese [46]. In the UAE, prevalence of obesity is 25% among men and 30.6% among women [47]. In Kuwait, a recent study (*n* = 3589 adults) reported that prevalence of obesity is 36.5% among men and 44% among women, while overweight affects 42% of men and 32.1% of women [48]. A systematic analysis in KSA in 2016 found that 27% and 40.23% of women are overweight and obese, respectively [49]. Another study from Al-Kharj region (*n* = 1019) reported that 54.3% were obese [50]. In Bahrain, the WHO estimated that 36.8% of adult women and 24.9% of adult men were obese in 2016 [51]. In Oman, 23.2% of adult men and 39.2% of adult women were obese in 2017 [52].

Obesity is a complex condition involving the integration of metabolic, genetic, and cultural factors. Nevertheless, the transition towards western lifestyles, which favor a high intake of energy-dense foods and sedentary, is the primary driver of obesity in GCC. The unprecedented economic growth played a significant role in this transition. Data show that 50.7% to 98.7% of GCC females do not exercise [45]. In 2007, a study assessed the levels of physical activity among Saudis (*n* = 17395, 30–70 years old) and found that 96% were physically inactive [53]. Bahraini women are also physically inactive; 93% walk less than 1 km per day [45]. Similarly, 71.6 to 80.8% of Kuwaiti women do not exercise [45]. In Qatar and Oman, rates of inactivity of 60.5% and 69.3% were documented [45]. In the UAE, 56.7% of women reported not walking for more than 20 minutes a day [54, 55].

Furthermore, teenagers exercise minimally in the GCC due to the overuse of digital gadgets and lack of playgrounds. People travel almost exclusively by car. In Dubai, the metro is used mostly by expatriates. It is not uncommon to see that even when people get food from fast-food restaurants, they do not step out of their cars, and food is delivered to their vehicles. Perhaps part of the blame falls on the scorching weather for about half a year, making it difficult even to walk outside, let alone exercise. Nevertheless, one might argue that, in sub-Saharan Africa, obesity is not as widespread as in the GCC; the most probable fault is the combination of a sedentary lifestyle and food abundance.

### 2.4. Reversing T2D: A Mission Not Impossible

Type 2 diabetes is regarded as a lifelong condition requiring constant vigilance from patients, families, and physicians. However, with a healthy lifestyle, combined or not with pharmacotherapy, hyperglycemia can be managed in many cases. Interestingly, the Diabetes Remission Clinical Trial (DiRECT) (*n* = 306, aged 20 to 65) has demonstrated that ∼50% of those with <6 years of T2D can return to long-term normoglycemia following a substantial and maintained weight loss (>5 kg) [56], suggesting that remission of T2D is possible. This trial showed that the remission of T2D varied ranging from 7% (0–5 kg weight loss) to 86% (≥15 kg). The DiRECT study relied on primary-care weight management, emphasizing that trained nurses and dietitians in primary healthcare centers could help people with T2D achieve the necessary weight loss. Results from the expected four-year follow-up of DiRECT participants are critical to examine the legacy impact of weight loss as postintervention regain of weight was recorded in most weight management studies [56, 57]. A first-in-kind recent study from Qatar reported that the ILI led to significant weight loss at 12 months and was associated with T2D remission in over 60% of young (18–50 years old), overweight or obese, and with short-duration T2D diabetes (≤3 years) participants [58]. This study is the first to investigate the impact of ILI on T2D in individuals from the MENA region and highlights once more the importance of early diagnosis of T2D and confirms the possibility to reverse T2D. Nevertheless, the long-term effect remains to be seen, as cultural factors influence attitudes towards weight loss and food, and the desire to achieve sufficient weight loss and, most significantly, to maintain it cannot be presumed.

### 2.5. Early Prediabetes Diagnosis: A Chance to Prevent T2D

Prediabetes and obesity are the primary risk factors for future T2D [59]. More often than not, obesity is generally viewed as a precursor to prediabetes, which is a precursor to T2D. The two factors are especially relevant to any strategy to curb the T2D epidemic. Therefore, screening for prediabetes in at-risk individuals should be strongly advocated, not only because it is a risk factor for T2D but also because it itself carries risks of comorbidities [60, 61]. Fortunately, recent research demonstrated that the progression to T2D could be prevented, or at least delayed, in a significant fraction of individuals with prediabetes. Hence, several prospective clinical trials have established the intensive lifestyle intervention (ILI) as one of the most effective strategies to prevent T2D when targeted to individuals with prediabetes [62]. For instance, in the prospective Diabetes Prevention Program (DPP), reversion to normal glycemia was associated with a significant 58% reduced risk of future T2D and even higher (71%) in subjects over 60 years of age. The Diabetes Prevention Program Outcomes Study (DPPOS) reported that the T2D incidence in 10 years was reduced by 34% in the ILI group, suggesting ILI's legacy for at least ten years [63]. Results from the same trial concluded that reversion to normoglycemia, even if transient, is associated with a significantly reduced risk of future diabetes [64]. The results were confirmed in other studies from Finland (58% risk reduction at 3.2 years) [65], China (42% risk reduction at 6 years) [66], and India (28.5% risk reduction at 3 years) [67]. In a late study (*n* = 8652), weight losses of 3–5% and >5% were associated with, respectively, 40% and 64% lower risk of developing T2D at six-year follow-up [68]. These studies support the idea that T2D incidence is preventable with lifestyle changes in people with prediabetes.

Studies to assess the effects of ILI on the reversion of T2D or the reduction of T2D risk in prediabetes patients are scarce in the Middle East region. Therefore, there is a lack of evidence regarding the effectiveness of ILI in GCC populations. However, Alfwaz and colleagues recently investigated ILI's effects on metabolic syndrome in Saudi adults with prediabetes (*n* = 294) [69]. They found that, at 12 months, the full metabolic syndrome decreased by 26% in the ILI group and only by 8.2% in the general advice group. Despite the short follow-up period and given the cultural and ethnic similarities between the GCC populations, this study demonstrates that ILI to prevent T2D may work in GCC populations. Moreover, the recent study by Taheri and his colleagues [58], showing ILI-induced T2D remission in 60% of participants, indicates that individuals with prediabetes in GCC might benefit from the ILI and with likely better remission rates.

### 2.6. Fighting Diabetes: The Time Is Now

Given the debilitating diabetes-associated complications, if the rates of obesity, T2D, and prediabetes in GCC are not reversed, not only will the region witness a rise in healthcare costs, but also its economic development will be at stake because of the decline in active populations. Considerable efforts are made to raise public awareness about diabetes health consequences and improve diabetes care across GCC. However, the message needs to be pushed incessantly across all social levels, from the government to the family. There is an urgency to formulate and implement effective and efficient approaches for (1) improving treatment of T2D patients, (2) reversing short-duration T2D, (3) reversing prediabetes, (4) fighting obesity in children and adults, and (5) upping the funding for diabetes and obesity research.

## 3. Improving the Treatment of T2D

**Figure 1:**
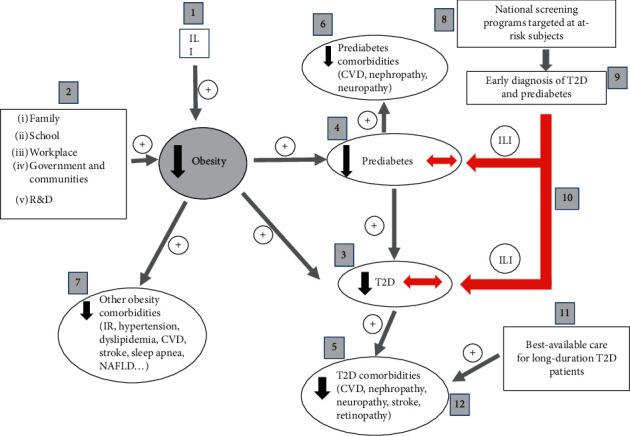
Concerted interventions required to curb diabetes and obesity epidemics. The battle against diabetes and obesity requires a holistic approach. Obesity rates can be reduced by intensive lifestyle intervention (ILI) (1) and prevented by concerted efforts between different partners, including family, school, workplace, government agencies, and research and development (2). Prevention of obesity will prevent and reduce the incidence of T2D (3) and prediabetes (4) and will ultimately prevent diabetes and prediabetes comorbidities ((5) and (6)). Prevention of obesity will also reduce other obesity-associated complications (7). On the other hand, national screening programs (8) targeted at at-risk individuals will permit the early identification of individuals with short-duration T2D and prediabetes (9). These subjects will be enrolled in ILI programs to try to reverse conditions (10). Finally, individuals with long-duration T2D need to be provided with the best available management and care (11) to prevent, or at least delay, T2D-associated complications (12). The downward black arrows indicate prevention and reduction of incidence. The red left-right arrows indicate reversion of the conditions. The + sign indicates a positive effect. ILI: intensive lifestyle intervention; T2D: type 2 diabetes; IR: insulin resistance; CVD: cardiovascular disease; NAFLD: nonalcoholic fatty liver disease.

Remission of short-duration T2D is now achievable in a significant fraction of patients by sustained weight loss (≥5% of body weight) [56, 58]. Unfortunately, reversion is not always possible because of late diagnosis (>6 years) or the difficulty of achieving and mainly maintaining necessary weight loss. In this situation, physicians need support to deliver the best available therapies to empower patients to control their glycemia tightly (Figure 1). In order to achieve these goals, T2D care should be best provided by multidisciplinary teams incorporating experts in management of diabetes and its comorbidities, nutrition, education, and exercise, as well as the patients' families [70, 71]. Successful management of T2D includes ILI, medications, discipline, monitoring for complications, and regular laboratory assessments. For better results, early diagnosis and well-designed and maintained national registries are crucial. In addition to epidemiology data, these registries enable the sharing of patient data across healthcare institutions and allow better monitoring and surveillance of patients and physicians' adherence to up-to-date treatments [72, 73]. Most of the GCC states have started establishing diabetes registries, but more work is needed. The GCC nations can learn from the Scandinavian countries whose registries are among the best in the world. Of note, these registries' success depends on efficient primary healthcare (PHC) facilities, and it is, therefore, imperative to strengthen and empower these facilities with sufficient funding and trained personnel. Diabetes registries are also essential for advancing diabetes research in the region.

## 4. Preventing and Reversing T2D

Current evidence supports the idea that T2D, if diagnosed at an early stage, could be reversed with ILI [56, 58, 74]. However, T2D returns in weight regain, highlighting the importance of tight surveillance for any successful T2D remission. The cornerstone of T2D remission is the early diagnosis (Figure 1). Therefore, regular checkups of at-risk citizens should be encouraged. Of note, the ADA recommends that adults aged ≥45 years are tested at least once every three years for T2D. However, earlier T2D onset has been reported for Middle Eastern Arabs [32, 75]. Thus, it is recommendable that regular checkups be carried out earlier than the age of 40 in the GCC region. The at-risk individuals should be taught about how lifestyle modification may prevent T2D.

Nevertheless, at the population level, the most pressing question remains: how to identify at-risk individuals? Some GCC countries have established national screening programs to address this question. In the UAE, Weqaya (“prevention” in Arabic) was launched in 2008 to understand noncommunicable diseases, particularly diabetes, better. To qualify for free health insurance coverage, all adult Emiratis must undergo annual cardiovascular risk screening. The program reported T2D or prediabetes in 44% of adult Emiratis (Health Authority of Abu Dhabi, Weqaya presentation 2009). Similarly, Qatar's national diabetes screening program was launched in 2018 by the Ministry of Public Health as part of the Qatar National Diabetes Strategy established in 2015. The program integrates public awareness and prevention activities, focusing on at-risk populations. In this regard, a pilot screening project, known as “the SMART clinic,” screened 3,500 individuals and found that 13% of them had T2D, and 21% had prediabetes. These initiatives highlight the importance of screening programs for the early diagnosis of T2D and prediabetes. Given the extent of the T2D epidemic in the GCC, governments could implement policies to somehow “compel” people to undergo regular checkups. Making these checkups a requirement for free health insurance coverage is one way of doing this, but other ways could be considered.

## 5. Fighting Obesity: “An Ounce of Prevention Is Worth a Pound of Cure”

The GCC is one of the regions affected the most by both childhood and adult obesity. The causative link between obesity and T2D is discussed elsewhere [76]. Nevertheless, given that weight loss reverses short-duration T2D and prediabetes, efforts to combat obesity, especially in youngsters, are crucial and require sustained commitment and public and private stakeholders' involvement. Different partners can play pivotal roles in battling the obesogenic environments, providing healthy surroundings and facilitating access to affordable, healthier dietary options (Figure 1). Some recommendations for the various players are discussed below.

### 5.1. Family

Every effort should be made at the family level to avoid sugar-rich drinks and snacks and to improve fruit, vegetables, wholegrain, and nuts intake. Restricting the average daily time spent on TV and digital devices, motivating children to exercise, and avoiding unhealthy sleep routines would help prevent obesity and achieve sustained weight loss in obese/overweight children. Parents should act as role models for their children, especially in healthy eating habits and exercising. They should also learn about the health risks of obesity and transmit this knowledge to their children.

### 5.2. Schools

Most youngsters in GCC are enrolled in schools, which are thus well positioned to have a considerable impact in the fight against obesity. Unfortunately, many schools are making insufficient efforts to prevent obesity, due to either a lack of resources or inadequate knowledge of obesity-related health issues. In partnership with education and finance ministries, health authorities in GCC should use the schools as an opportunity to raise awareness about obesity among kids at an early age. Schools could (1) invite obesity experts and dietitians to teach students and parents about obesity health risks, nutrition literacy, and healthy lifestyle. In fact, given the magnitude of the problem, nutrition literacy should be woven into core classroom subjects from an early age to teach pupils skills to maintain a healthy lifestyle. They could (2) increase the hours of physical education to provide the kids the opportunity to improve the fitness, socialize, increase concentration and self-esteem, better academic scores, build a healthier body, improve posture and balance, lower stress, and encourage a better night's sleep. They could (3) surround students with opportunities to eat healthily and to stay active outside the classroom. Calorie-dense foods and drinks should be prohibited from vending machines and in school canteens. Instead, school cafeterias should include healthier and affordable food offerings and prohibit marketing unhealthy foods.

Foods packed within school lunchboxes may contribute to energy imbalance [77]. In this context, Qatar Academy has implemented a policy that forbids lunchboxes, except for medical reasons. Similar measure could be implemented in all GCC schools. They could also (4) empower and train school nurses to conduct routine checkups to identify overweight/obese kids and those with prediabetes or diabetes. In brief, schools must be supported to be an integral part of the fight against the obesity epidemic by helping and teaching students to eat better, be more active, and achieve healthier weights.

### 5.3. Workplace

The workplace is an environment filled with elements that can promote or diminish health [78]. Obesity-associated comorbidities can have a massive impact on workers and corporate health by undermining productivity, worsening mobility and morale, and increasing absenteeism and occupational injuries [79, 80]. The workplace can be an optimal and productive environment for improving health and combating obesity. Companies in the GCC should be made aware of and encouraged to provide employees with relevant education, skills, and resources to facilitate healthier diets and physical activity. Programs can include nutrition courses, access to dietitians, provision of healthier food options in on-site canteens, and exercise facilities or incentives such as reimbursement of exercise-related expenses. Signs to use stairs instead of lifts and to take a short walk every one-to-two hours could prevent sedentary lifestyle.

### 5.4. Government and Communities

The creation of healthy environments (i.e., places to keep people moving) is another area that GCC authorities might focus on to fight obesity. The surroundings where people live and work could strongly impact their actions, particularly when it comes to physical activity. If a neighborhood lacks sidewalks, few people will walk. It is unfortunate, indeed, to notice that pavements are still in short supply in most cities in GCC, and walking to work could be a dangerous adventure. There is also an almost complete lack of bicycle lanes for people willing to cycle to work, school, park, or mall. GCC countries can learn from countries like Netherlands, where there is a national cycle network made of many safe bicycle lanes, and some 36% of people use bicycles as their frequent mode of transport [81]. Culture and hot weather could be an obstacle for persuading people to use bicycles in this area of the world. However, if safe lanes are provided and the health benefits of bicycling are highlighted in mass media, we may see a gradual change in the future. There are also a few and remote safe public places to exercise. Local authorities should invest more in local parks for kids and adults to work out or play their preferred sports.

Finally, extreme measures by the GCC governments, such as imposing high taxes on sugar-sweetened beverages and fast-foods, should be considered. The taxes generated could be injected back into raising public awareness and fighting obesity. Limiting the number of fast-food restaurants per surface area and placing strict regulations on the marketing of unhealthy foods, particularly to children, are other measures that might help in the fight against obesity. In short, preventing obesity in the GCC requires the involvement and commitment of several public and private stakeholders and is the key to curb the T2D epidemic.

### 5.5. Research and Development

All the GCC countries now have visions and objectives to use natural resources and transform their traditional economies to more diversified, knowledge-based, and sustainable ones. At the heart of these endeavors lie the critical components of research and development, technology, knowledge, and innovation. In the context of fighting diabetes and obesity, research and development (R&D) constitute an essential pillar. Unfortunately, despite some promising initiatives that started to immerge during the last decade, the GCC states are lagging in R&D. In Qatar, since 1995, the Qatar Foundation for Education, Science and Community Development has invested heavily in research and funded work in a wide range of fields, including biomedical research. Concerning diabetes and obesity, the Qatar Foundation funds programs aimed at improving and developing new therapies and basic and translational work related to diabetes prevention and understanding of pathophysiology and genetics. Several research institutes working under the Qatar Foundation umbrella, as well as the Hamad Medical Corporation and the primary healthcare corporations, and different colleges in Qatar University are actively involved in diabetes and obesity research in the country. In Kuwait, the Dasman Diabetes Institute (DDI) was established to address the country's growing diabetes epidemic. Likewise, in the UAE, the Dubai Diabetes Center and the Imperial College London Diabetes Center were established to improve people with diabetes and conduct diabetes research. University research groups and hospitals across the GCC are also contributing to different research initiatives. Although there is a long way to go, it is imperative to have research funding agencies that put diabetes and obesity as a top health priority. Given that the GCC's research culture is still in its infancy, it is of paramount importance that research funding is upped and sustained, and the logistics and regulations are implemented to support researchers at all levels.

## 6. Conclusion and Future Perspectives

The GCC nations witness unprecedented obesity and diabetes epidemics. The rates are startling, and the projections look bleak without immediate actions. Obesity is recognized as the primary driver of the soaring diabetes rates in GCC. In turn, obesity is driven by an obesogenic environment consisting of sedentary lifestyle, rapid urbanization, and an abundance of calorie-dense foods. The cost of the care of diabetes and obesity and their associated complications is stretching the health budgets in all GCC nations. Further, their burden will also weigh heavily on the broader economy due to loss of productivity. Tackling diabetes and obesity should involve several public and private stakeholders, and recommendations to curb the two epidemics may include (1) boosting healthcare investment to improve the treatments; (2) strengthening primary healthcare facilities with motivated and qualified personnel; (3) introducing large-scale screening programs for early identification of T2D and prediabetes; (4) taking a cross-governmental approach to reduce obesity, the primary cause of diabetes; (5) introducing strict and stringent new legislation to regulate unhealthy foods and drinks; and (6) investing in research to better understand the pathophysiology of diabetes and obesity in the GCC region and identify new ways and new biological markers to predict the diseases and prevent them from happening in the first place.

## Data Availability

No data were used in this study.

## Abbreviations

DDI: Dasman Diabetes Institute
GCC: Gulf Cooperation Council
KSA: Kingdom of Saudi Arabia
ILI: Intensive lifestyle intervention
MENA: Middle East and North Africa
T2D: Type 2 diabetes
UAE: United Arab Emirates.
